# Tuberin haploinsufficiency is associated with the loss of OGG1 in rat kidney tumors

**DOI:** 10.1186/1476-4598-7-10

**Published:** 2008-01-24

**Authors:** Samy L Habib, Simona Simone, Jeff J Barnes, Hanna E Abboud

**Affiliations:** 1 O'Brien Kidney Research Center, Department of Medicine, University of Texas Health Science Center, San Antonio, TX 78229, USA; 2 South Texas Veterans Healthcare System, Geriatric Research, Education, and, Clinical Center, San Antonio, Texas 78229, USA; 3 Department of Emergency and Transplantation, University of Bari, Policlinico, Bari, Italy

## Abstract

**Background:**

Tuberous sclerosis complex (TSC) is caused by defects in one of two tumor suppressor genes, *TSC-1 *or *TSC-2*. *TSC-2 *gene encodes tuberin, a protein involved in the pathogenesis of kidney tumors. Loss of heterozygosity (LOH) at the *TSC2 *locus has been detected in *TSC*-associated renal cell carcinoma (RCC) and in RCC in the Eker rat. Tuberin downregulates the DNA repair enzyme 8-oxoguanine DNA-glycosylase (OGG1) with important functional consequences, compromising the ability of cells to repair damaged DNA resulting in the accumulation of the mutagenic oxidized DNA, 8-oxo-dG. Loss of function mutations of OGG1 also occurs in human kidney clear cell carcinoma and may contribute to tumorgenesis. We investigated the distribution of protein expression and the activity of OGG1 and 8-oxo-dG and correlated it with the expression of tuberin in kidneys of wild type and Eker rats and tumor from Eker rat.

**Results:**

Tuberin expression, OGG1 protein expression and activity were higher in kidney cortex than in medulla or papilla in both wild type and Eker rats. On the other hand, 8-oxo-dG levels were highest in the medulla, which expressed the lowest levels of OGG1. The basal levels of 8-oxo-dG were also higher in both cortex and medulla of Eker rats compared to wild type rats.

In kidney tumors from Eker rats, the loss of the second *TSC2 *allele is associated with loss of OGG1 expression. Immunostaining of kidney tissue shows localization of tuberin and OGG1 mainly in the cortex.

**Conclusion:**

These results demonstrate that OGG1 localizes with tuberin preferentially in kidney cortex. Loss of tuberin is accompanied by the loss of OGG1 contributing to tumorgenesis. In addition, the predominant expression of OGG1 in the cortex and its decreased expression and activity in the Eker rat may account for the predominant cortical localization of renal cell carcinoma.

## Background

Oxidative DNA damage is one of the most common threats to genomic stability; DNA repair enzymes provide protection from the effects of oxidized DNA bases. Mutations that influence the repair of oxidized DNA modifications are expected to increase the steady-state (background) levels of these modifications and thus create a mutator phenotype that predisposes to malignant transformation [1-3]. Many of these mutations occur as a result of irreparable or incompletely repaired genomic DNA, which is constantly subject to assault from intrinsic and environmental insults. Oxidized forms of DNA in particular are produced in mammalian cells as a byproduct of normal oxidative metabolism or in response to exogenous sources of reactive oxygen species [4,5]. 8-Oxo-deoxyguanine (8-oxodG) is one of the major base lesions formed after oxidative damage to DNA [6,7]. 8-oxodG is mutagenic since it pairs with adenine during DNA synthesis, increasing G:C to T:A transversions [8]. Oxidative damage-induced mutations activate oncogenes or inactivate tumor suppressor genes, altering cell growth control. 8-oxodG in DNA is repaired primarily via the DNA base excision repair pathway [9]. The gene encoding the DNA repair enzyme that recognizes and excises 8-oxodG is 8-oxoG-DNA glycosylase (OGG1) [10]. Deficiency of OGG1 in yeast, or its homologue formamidopyrimidine-DNA glycosylase in bacteria, results in a spontaneous mutator phenotype [11]. The steady-state levels of 8-oxoG, which reflect the balance between continuous generation and removal, are significantly higher in livers of *OGG1*^-/- ^mice compared to wild-type animals [12]. The *OGG1 *gene is somatically mutated in some cancer cells and is highly polymorphic among humans [13,14]. Loss of heterozygosity at the *OGG1 *allele, located on chromosome 3p25, was found in 85% of 99 human kidney clear cell carcinoma samples, identifying the loss of OGG1 function as a possible consequence of multistep carcinogenesis in the kidney [15].

Tuberous sclerosis complex (TSC) is a genetic disorder associated with tumors in many organs, including renal cell carcinoma. It affects about 1 million individuals worldwide, with an estimated prevalence of up to one in 6,000 newborns [16]. Loss of heterozygosity (LOH) at *TSC2 *locus has been detected in *TSC*-associated renal cell carcinoma (RCC) [17,18]. The Eker rat has been used for many years to model TSC and RCC. In this strain, the incidence of clear cell RCC in gene carriers approaches 100% by 1 year of age [19,20]. The constitutive expression of OGG1 in heterozygous Eker rat (TSC2^+/-^) kidneys is lower than in wild-type rats [21] suggesting that these proteins may be functionally linked. The present study was conducted to investigate the basal levels and localization of tuberin, 8-oxodG and OGG1 in different regions of wild type and Eker rat kidneys.

## Results

### Distribution of tuberin and OGG1 in kidney

**Figure 1:**
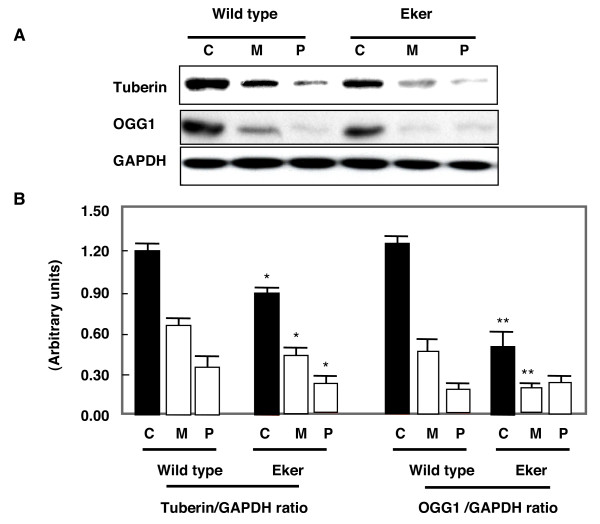
Distribution of tuberin and OGG1 protein in kidney cortex (C), medulla (M) and papilla (P) of wild type and Eker rats. A. Immunoblot analysis of tuberin and OGG1 in different kidney regions. Different kidney regions were homogenized and protein extracts were loaded onto 7% SDS-polyacrylamide gels and transfered to PVDF membrane. The membrane was incubated with anti-tuberin or anti-OGG1 followed by different specific HRP-conjugated secondary antibodies. The proteins were visualized by ECL. GAPDH was used as loading control. B. Histograms represent means ± SE (n = 3). Significant difference from wild type rat is indicated by * P < 0.05 and ** P < 0.01.

Kidney homogenates from cortex, inner medulla and papilla were subjected to western blot analysis. As expected, tuberin expression in kidney of wild type animals was higher than in Eker rats. Kidney cortex including the outer medulla shows higher tuberin and OGG1 expression compared to inner medulla and papilla. Deficiency of tuberin in Eker rats was associated with decrease in OGG1 expression compared to wild type rat in all three regions of the kidney (Fig. 1A).

### Decrease in OGG1 activity is associated with increases 8-oxodG in Eker rat

**Figure 2:**
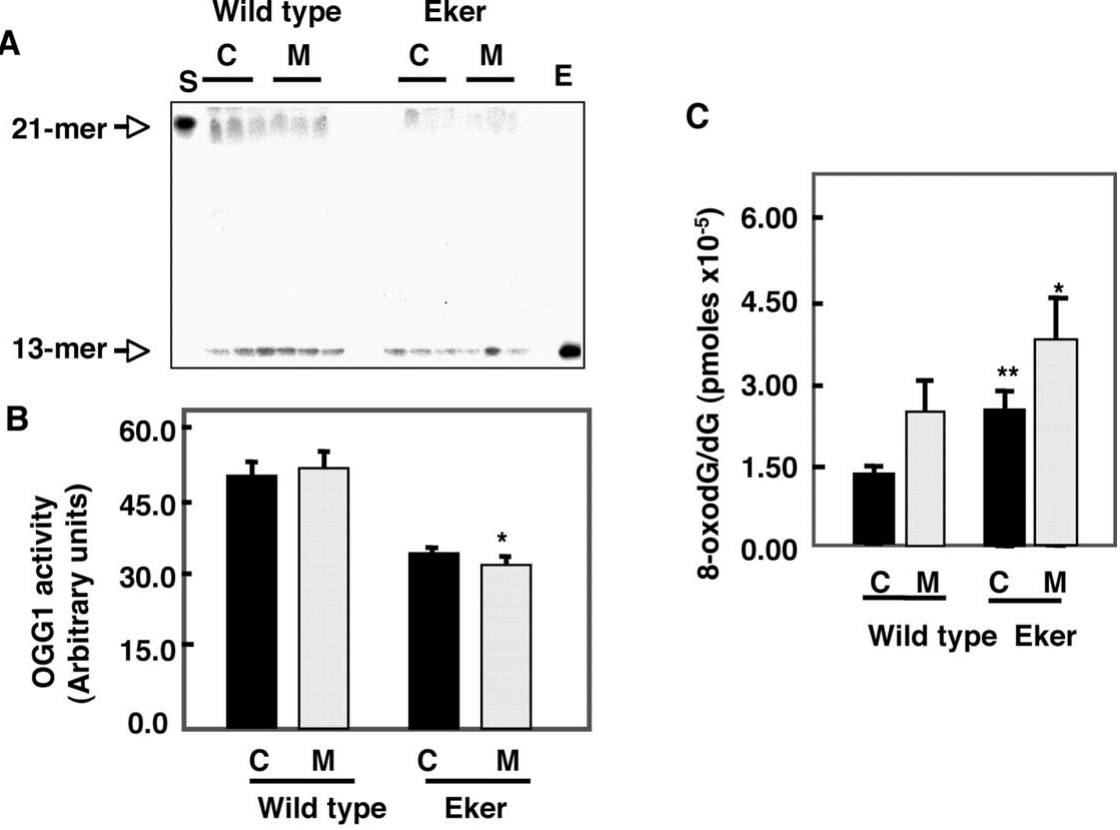
Distribution of renal OGG1 activity and 8-oxodG in cortices (C) and medulla (M) of wild type and Eker rat. A. 21-mer containing an 8-oxoG lesion was labeled at its 5' end using [32P] ATP and incubated with cortex and medulla kidney homogenate of wild type and Eker rat. Oligonucleotide cleavage products were analyzed on DNA sequencing gels and subjected to autoradiography. Pure human OGG1 enzyme (E) and buffer alone (S) were analyzed as positive and negative controls, respectively. The top arrow indicates the 21-mer of 8-oxodG as a substrate and the bottom arrow is the DNA cleavage product (13-mer). B. Histograms represent means ± SE (n = 3). C. DNA was extracted and digested with nuclease P1. The detection of dG and 8-oxodG was performed by HPLC-EC analysis. Authentic standards of 8-oxodG and dG were analyzed simultaneously. Standard curves for dG and 8-oxodG were prepared and quantitated by linear regression analyses. Significant difference from wild type rat is indicated by * *P *< 0.05 and ** *P *< 0.01.

To determine if the decreases in OGG1 protein expression correlates with the enzymatic activity, OGG1 activity was compared in kidneys from wild type or Eker rats. DNA glycosylase activity of the OGG1 enzyme was assayed as the cleavage of an 8-oxoG-containing oligomer, which releases oxidized guanine base from a ^32^P-labeled 21 oligonuleotide. OGG1 activity was higher in cortex compared to the medulla (Fig. 2A). OGG1 activity was significantly lower in kidney cortex and medulla of Eker rats compared to wild type rats (Fig. 2B). OGG1 is the major DNA base excision repair enzyme that recognizes and excises 8-oxodG, therefore we determined whether the change in OGG1 abundance influenced the accumulation of 8-oxodG, a substrate of OGG1 enzyme. In order to evaluate the basal level of oxidative DNA damage in the cortices and inner medullae of Eker and wild type rats, we examined the content of 8-oxo-dG in the nuclear DNA extracted from both section using HPLC-EC. DNA was isolated from kidney cortices and medulla of Eker and wild type rats and 8-oxodG levels were analyzed by HPLC. 8-OxodG levels were higher in kidney tissue from Eker compared to wild type rats (Fig. 2C). The levels of 8-oxodG in nuclear DNA were significantly higher (2 fold) in cortices of Eker compared to wild type rat. The levels of 8-oxodG were significantly higher in kidney medullae compared to kidney cortices. These data suggest that OGG1 protein and activity express the low levels in inner medullae may not be sufficient to repair the generated 8-oxodG compared to cortex region.

### Histology of kidney tumor in Eker and wild type rat

**Figure 3:**
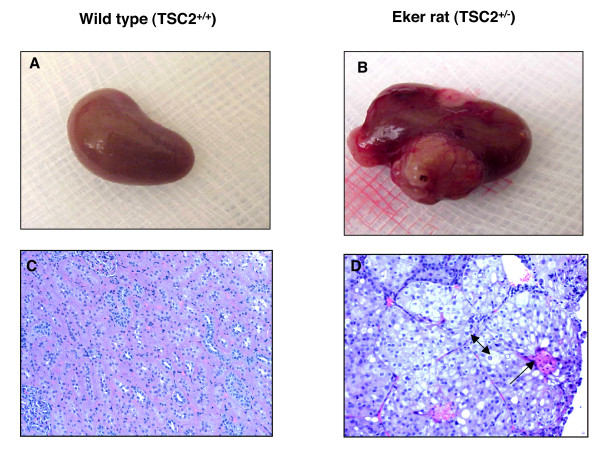
(A) Kidney from wild type and (B) Eker rats were harvested at 12 months. Kidney from wild type rat show normal size and appearing while large size and multiple tumors were appeared in kidney of Eker rat. H&E staining of kidney from (C) wild type and (D) Eker rats. Kidney section of wild type and Eker rats shows normal tubular architecture of kidney in wild type rat and fibrovascular stroma (indicated by one head arrow) with clear cytoplasm and excentric in large nuclei of clear cell carcinoma type (indicated by two head arrow) in Eker rat.

Kidney from wild type and Eker rats were harvested at 12 months of age. Kidneys from wild type rats showed normal size and appear in contrast to Eker rats that larger kidneys with multiple tumors (Fig. 3A &3B). The histological appearance of kidney sections of wild type and Eker rats by H& E staining showing normal tubular architecture of kidney in wild type rat. However, Eker kidneys showed fibrovascular stroma and tumors containing cells with clear cytoplasm and excentric in large nuclei of clear cell carcinoma type (Fig. 3C &3D).

### Localization of tuberin and OGG1 in the kidney

**Figure 4:**
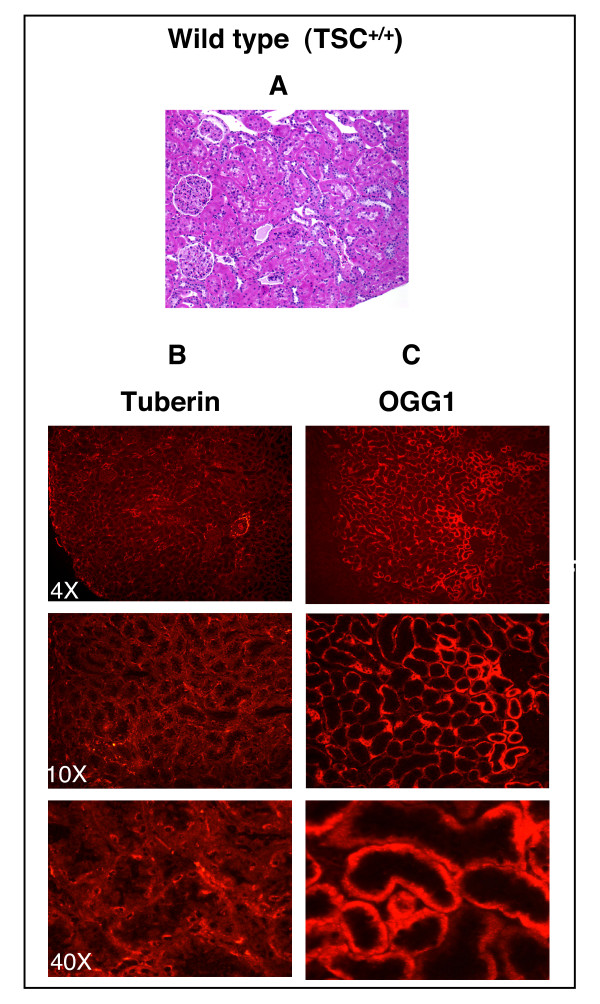
Tuberin and OGG1 staining are mainly localized in the kidney cortex. (A) H&E staining in kidney section of wild type rats (*TSC2*^+/+^) show normal tubular architecture. The majority staining of tuberin (B) and OGG1 (C) are localized in the cortical region compared to medulla region.

**Figure 5:**
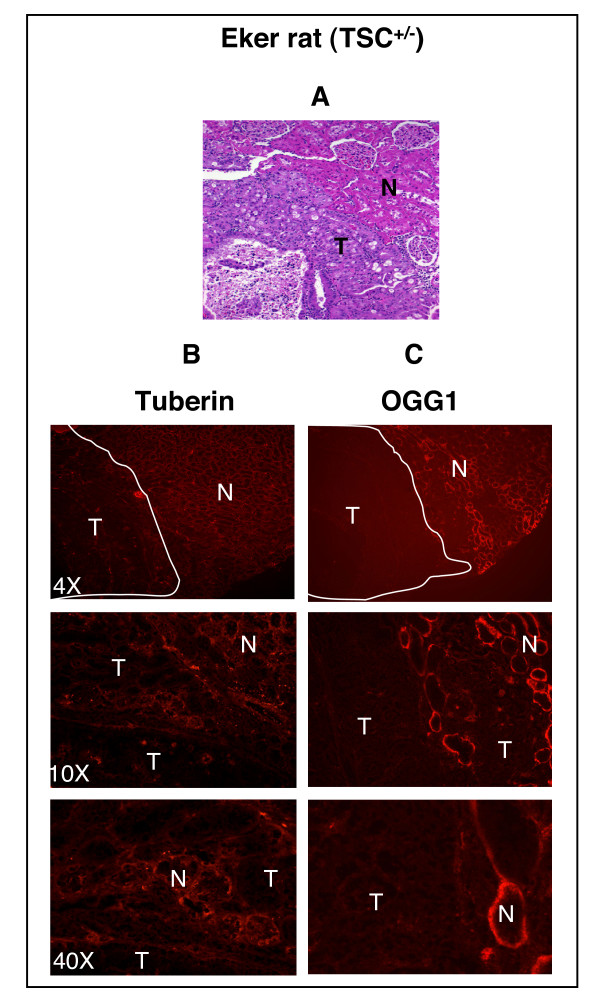
Loss of tuberin is associated with loss of OGG1 in kidney tumor of Eker rat. (A) H&E staining in kidney section of Eker rats (*TSC-2*^+/-^) show cortical tumors. The tuberin (B) and OGG1 (C) staining are not detected in tumor (T) compared to normal tissue (N) of cortical region.

H& E staining shows normal tubular architecture of the kidney in cortex and medulla of wild type rat (Fig. 4A). Tumors are seen clearly in the cortical region of the kidney of Eker rat (Fig. 5A). Immunohistochemistry and western blot were performed to determine the association between the expression of tuberin and OGG1 in Eker and wild type rats kidney sections. Tuberin is expressed in many cell lines and tissues including adult rat kidney. Tuberin staining was higher in the cortex than in the medulla and specifically in interstitial cells and more predominantly in the  collecting duct and distal tubules in both wild type and Eker rats (Fig. 4B &5B). Other regions of the nephron such as the glomerulus revealed no tuberin staining. Tuberin staining was higher in wild type kidney and less in kidney tumor from Eker rat (Fig. 4B &5B). OGG1 staining was higher in the cortex than in the medulla in both wild type and Eker rat kidneys (Fig. 4C &5C). At a higher magnification, a substantial level of OGG1 was noted in the distal and proximal convoluted tubules of the cortex and negative staining observed in the glomerular (Fig. 4 &5C). OGG1 staining was higher in cortices and the outer medullae than in inner medullae in both wild type and Eker rat kidneys (Fig. 4C &5C) while was almost not detected in kidney tumor from Eker rat (Fig. 5C).

### Loss of tuberin is associated with loss of OGG1

**Figure 6:**
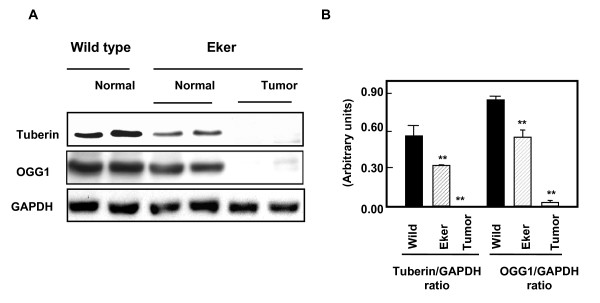
A deficiency in tuberin is associated with loss of OGG1 expression in Eker rat kidney tumors. A. Immunoblot analysis of tuberin and OGG1 protein expression in normal kidney of wild type rats, normal and tumor kidney tissue from Eker rats. GAPDH was used as loading control. B. Histograms represent means ± SE (n = 3). Significant difference from wild type rat is indicated by ** *P *< 0.01.

To confirm if the loss of tuberin is associated with loss of OGG1 expression in tumor of Eker rat, normal and tumor kidney tissue from Eker rat as well as normal kidney from wild type rat was examined by western blot analysis. Normal appearing tissue of kidney cortex shows decrease in tuberin expression associated with decrease in OGG1 protein expression (Fig. 6). Tumor homogenate from cortex kidney of Eker rat show null tuberin and OGG1 expression (Fig. 6) indicating that tuberin is an upstream regulator of OGG1 protein and gene expression. These results are consistent with the data obtained by immunostaining analysis in Figures 4 &5 indicating that tuberin is upstream of OGG1.

## Discussion

This study is the first report that the loss of tuberin is associated with loss of OGG1 suggesting that both proteins may play a major role in development of kidney tumor in Eker rat. Our data demonstrate that tuberin deficiency is associated with deficiency of protein as well as with marked inhibition of OGG1 activity indicating that tuberin is upstream of OGG1. In addition, our data show that inhibition of OGG1 activity is associated with increased accumulation of 8-oxodG in kidneys of Eker compared to wild type rats. We have show that the constitutive expression of OGG1 in TSC2 heterozygous Eker rat (TSC2^+/-^) kidneys is lower than in wild-type rats [21]. The protein and activity levels of OGG1 were higher in cortical region than in the medullary region of wild type rat. However, OGG1 protein and activity were lower in kidney cortices and medullae of Eker compared to wild type rats. In addition, 8-oxodG accumulation was significantly higher in kidney medulla compared to kidney cortex indicating that inner medulla could express low level of DNA repair OGG1 that not sufficient to repair the amount of 8-oxodG compared to cortex region. OGG1 expression was low in kidney of Eker rat and that associated with high amount of 8-oxodG compared to wild rat suggesting that Eker rats are more susceptible to oxidative DNA damage. In addition, tumor kidney of Eker rat show null tuberin and OGG1 expression indicating that tuberin is an upstream regulator of OGG1 protein and gene expression. Furthermore, the kidney has an unusually high rate of oxygen metabolism, and it has been shown that the high concentrations of urea frequently present in kidney cells trigger oxidative stress [15] and therefore increase the likelihood of DNA damage by 8-oxodG formations.

In mammalian cells, the base excision pathway initiated by OGG1 represents the main defense against the mutagenic effects of 8-oxodG. However, the decrease in OGG1 expression in *Tsc-2*^+/- ^rats has important functional consequences, compromising the ability of these animals to respond to oxidative stress. Indeed, the loss of OGG1 expression in kidney tumor tissue from Eker rat resulted in the accumulation of significant amounts of 8-oxodG (unrepaired DNA lesions), suggesting that loss of tuberin was biologically significant in affecting OGG1. Treatment of Eker rats with an oxidative DNA damaging agent increases 8-oxodG formation [21], but the accumulation of 8-oxodG in wild type and Eker rats in our studies occurs in the absence of exposure to exogenous oxidants. 8-OxodG induces mutation via misincorporation of DNA bases present in the unrepaired DNA adducts, or by slippage of DNA polymerase during replicative bypass. We have recently shown that the basis for the suppression of renal OGG1 in tuberindeficient cells is sufficient to downregulate OGG1, at least in part through the transcription factor, NF-YA, the major transcription factor regulate OGG1 activity, with consequent decreased transcription of OGG1 [22].

The OGG1 protein initiates the base excision repair process by recognizing and excising the modified base. Consistent with the yeast findings, mice lacking a functional OGG1 protein accumulate abnormal levels of 8-oxoG in their genomes and display a moderately elevated spontaneous mutation rate in nonproliferative tissues [12]. Because inactivation of the *OGG1 *gene in mammalian cells causes a mutator phenotype, it can be expected that cells lacking OGG1 activity could have an enhanced probability of undergoing malignant transformation [23]. The validation of this hypothesis requires the identification of human tumors where both alleles of the *OGG1 *gene are nonfunctional. The human *OGG1 *gene is located on chromosome 3p25. Human chromosome 3p cytogenetic abnormalities and LOH have been observed at high frequency in sporadic forms of RCC. The 3p21.2-p21.3 locus is frequently deleted in RCC and also in other cancers, but to date, no candidate gene has been identified.

In summary, deficiency of tuberin is associated with loss of function of OGG1 in tumors of Eker rat. Impaired OGG1 repair enzyme activity results in the accumulation of 8-oxodG, suggesting that tuberin plays a significant role in protecting the cells from oxidative DNA damage. We propose that loss of OGG1 expression and function in tuberin-deficient tumor tissue predisposes to further genetic alterations as a result accumulation of mismatched DNA base lesions, a form of genomic instability that if left unrepaired promotes additional genetic alterations leading to the full blown tumor phenotype in kidney patients with TSC.

## Conclusion

The protein and activity levels of OGG1 were higher in cortical region than in the medullary region of wild type rat. However, OGG1 protein and activity were lower in kidney cortices and medullae of Eker compared to wild type rats. 8-Oxo-dG levels were highest in the medulla, which expressed the lowest levels of OGG1. The basal levels of 8-oxo-dG were also higher than in both cortex and medulla of Eker rats compared to wild type rats. OGG1 expression was low in kidney of Eker rat and that associated with high amount of 8-oxodG compared to wild rat suggesting that Eker rats are more susceptible to oxidative DNA damage. In addition, tumor kidney of Eker rat show null tuberin and OGG1 expression indicating that tuberin is an upstream regulator of OGG1 protein and gene expression.

## Materials and Methods

### Animals

Wild type (*TSC-2*^+/+^) and Eker male rats (mutant *TSC-2*^+/-^) were purchased from a breeding colony maintained at the University of Texas MD Anderson Cancer Center, Smithville, TX. The animals were allowed food and water *ad libitum *throughout the experiments. Animals were euthanized at 2 and 12 months for nephrectomy. The kidneys were dissected longitudinally, half preserved in 10% formalin in PBS, pH 7.4 for immunostaining and the remaining tissue was used for biochemical assays. Cortex, outer stripe of the outer medulla (OSOM) and papilla of the kidney were excised and frozen immediately in liquid nitrogen for biochemical analysis.

### Histology of normal and tumor kidney

Formalin-fixed kidneys were embedded in paraffin, 3 μm sections stained with hematoxylin and eosin for histological examination by light microscopy.

### Protein extraction and immunoblot analysis

Homogenates of kidney cortex, medulla and papilla were prepared in lysis buffer (1× PBS, 1% NP-40, 0.5% sodium deoxycholate, 0.1% SDS) containing phenylmethylsulfonyl fluoride (10 mg/ml), leupeptin (10 mg/ml), and aprotinin (20 mg/ml). Tissue homogenates was centrifuged at 14,000 × *g *for 30 min at 4°C. Protein concentrations were determined with the Bradford assay [24] using bovine serum albumin as a standard. Protein (100 μg) was subjected to SDS-polyacrylamide gel electrophoresis, transferred to polyvinylidene difluoride (PVDF) membranes and blocked in 5% nonfat dried milk in TBS- 0.1% tween buffer [25 mM Tris-HCl, 0.2 mM NaCl; 0.1% Tween 20 (v/v) pH 7.6] (TBS-T) for 1 hr. Membranes were incubated with the respective primary antibodies overnight at 4°C. Anti-OGG1 antibody was generously provided by Dr. S. Mitra [25] and anti-tuberin was purchased from Santa Cruz, CA. Membranes were washed 5× with TBS-T, and then incubated with an appropriate HRPconjugated secondary antibody for 1 h at RT. An enhanced chemiluminescence kit (Amersham, NJ) was used to identify the protein expression. Membranes were stripped with 0.2 M NaOH for 10 min each, blocked with 5% milk for 1 h, and then incubated with GAPDH (Santa Cruz, CA) as loading control. Expression of each protein was quantified by densitometry using National Institutes of Health Image 1.62 software and normalized to a loading control.

### Immunostaining of OGG1 and tuberin

Localization of OGG1 and tuberin was assessed by immunofluorescence histochemistry as previously described [26]. Acetone-fixed frozen sections (6 μm) were incubated with nonimmune donkey IgG to block nonspecific binding, then incubated with rabbit anti-tuberin (C-20, Santa Cruz Biotech) or OGG1 primary antibodies followed by FITC- or Cy3-labeled donkey anti-rabbit IgG (Chemicon International, Inc., Temecula, CA, USA) as secondary antibodies for signal detection. All incubations of primary and secondary antibodies were for 30 minutes with three washes with phosphate-buffered saline (PBS) containing 0.1% bovine serum albumin (BSA), 5 minutes each between steps. Controls consisted of non-immune mouse IgG or PBS/BSA in place of primary antibody followed by detection procedures as outlined above. Sections were viewed and photographed using an Olympus Research microscope equipped for epifluorescence with excitation and band pass filters optimal for either FITC or Cy3.

### Analysis of OGG1 activity

Kidney tissue was homogenated in 0.25 ml lysis buffer (20 mM Tris-HCl pH 8.0, 1 mM EDTA, 250 mM NaCl, 0.8 μg/ml antipain, 0.8 mg/ml leupeptin, 0.8 μg/ml aprotinin). The homogenate was sonicated for 8 sec at 4°C with pulses of 1 sec each between 10 sec intervals. After centrifugation (12,000 rpm) at 4°C for 15 min, the supernatant was recovered and protein content was determined by Bradford method (242). A 24-mer oligonucleotide containing the oxidized G base (R&D Systems, Inc, MN) [27], was labeled at its 5' end using [32P] ATP and T4 polynucleotide kinase. The 32P-labeled strand was hybridized with the complementary oligonucleotide A by incubation at 90°C for 10 min followed by slow cooling to RT. The assay mixture (20 μl final volume) contained 50 fmol of 32P-labelled DNA duplex and cell extracts (50 μg of protein) in 1× REC buffer (10 mM HEPES-KOH, pH 7.4, 100 mM KCL, 10 nM EDTA, and 0.1 mg/ml BSA). Purified OGG1 enzyme was used as positive control. The reactions were performed at 37°C for 1 h. Ten μl of 3× alkali loading buffer (300 mM NaOH, 97% formamide, and 0.2% bromophenol blue) was added, the samples heated at 95°C for 10 min and then fast cooled to 2–8°C. The cleavage products were resolved by 20% denaturing PAGE in the presence of 7 M urea.

### 8-OxodG assay

DNA was isolated from rat kidney tissue and detection of dG and 8-oxodG was performed on DNA hydrolyzed with nuclease P1 and alkaline phosphatase as previously described and validated [21]. Aliquots (90 μl) of DNA hydrolysates were injected onto a Partisil 5 μm ODS-3 reverse-phase analytical column for HPLC analysis with the eluate monitored with a UV photodiode array (Shimadzo, SPD M10A) and electrochemical (EC) detectors (ESA Coul Array). Authentic standards of 8-oxodG and dG were analyzed along with every batch of samples. Salmon sperm DNA (5–50 μg) was used as a positive control for DNA digestion reactions. Standard curves for dG and 8-oxodG were prepared and quantitation performed by linear regression analyses. Data were expressed as pmol 8-oxodG/dG × 10-5 in 90 μl of DNA hydrolysate.

### Statistics

Data are presented as mean ± standard error. Statistical differences were determined using ANOVA followed by Student Dunnett's (Exp. vs. Control) test using 1 trial analysis. *P *values less than 0.05 and 0.01 were considered statistically significant.

## Abbreviations

TSC2, tuberous sclerosis complex-2; 8-oxodG, 8-oxodeoxyguanine; OGG1, 8-oxoG-DNA glycosylase, RCC, renal cell carcinoma.

## Competing interests

The author(s) declare that they have no competing interests.

## Authors' contributions

SLH and HEA conceived the concept, designed the study, and prepared the manuscript. SS performed the western blotting. JB performed the immunohistochemistry and the pathological analyses of the animal tissue samples. All authors have read and approved the manuscript.

## References

[B1] Loeb LA (1991). Mutator phenotype may be required for multistage carcinogenesis. Cancer Res.

[B2] Chevillard S, Radicella JP, Levalois C, Lebeau J, Poupon MF, Oudard S, Dutrillaux B, Boiteux S (1998). Mutations in *OGG1*, a gene involved in the repair of oxidative DNA damage, are found in human lung and kidney tumours. Oncogene.

[B3] Shinmura K, Kohno T, Kasai H, Koda K, Sugimura H, Yokota J (1998). Infrequent mutations of the *hOGG1 *gene, that is involved in the excision of 8-hydroxyguanine in damaged DNA, in human gastric cancer. Jpn J Cancer Res.

[B4] Nishigori C, Hattori Y, Toyokuni S (2004). Role of reactive oxygen species in skin carcinogenesis. Antioxid Redox Signal.

[B5] Shi X, Castranova V, Halliwell B, Vallyathan V (1998). Reactive oxygen species and silicainduced carcinogenesis. J Toxicol Environ Health B Crit Rev.

[B6] Sakumi K, Furuichi M, Tsuzuki T, Kakuma T, Kawabata S, Maki H, Sekiguchi M (1993). Cloning and expression of cDNA for a human enzyme that hydrolyzes 8-oxo-dGTP, a mutagenic substrate for DNA synthesis. J Biol Chem.

[B7] Wilson DM, Bohr VA (2007). The mechanics of base excision repair, and its relationship to aging and disease. DNA Repair (Amst).

[B8] Cheng KC, Cahill DS, Kasai H, Nishimura S, Loeb LA (1992). 8-Hydroxyguanine, an abundant form of oxidative DNA damage, causes G----T and A----C substitutions. J Biol Chem.

[B9] Smart DJ, Chipman JK, Hodges NJ (2006). Activity of OGG1 variants in the repair of prooxidant-induced 8-oxo-2'-deoxyguanosine. DNA Repair (Amst).

[B10] Mitra S, Boldogh I, Izumi T, Hazra TK (2001). Complexities of the DNA base excision repair pathway for repair of oxidative DNA damage. Environ Mol Mutagen.

[B11] Guibourt N, Boiteux S (2000). Expression of the Fpg protein of Escherichia coli in Saccharomyces cerevisiae: effects on spontaneous mutagenesis and sensitivity to oxidative DNA damage. Biochimie.

[B12] de Souza-Pinto NC, Eide L, Hogue BA, Thybo T, Stevnsner T, Seeberg E, Klungland A, Bohr VA (2001). Repair of 8-oxodeoxyguanosine lesions in mitochondrial DNA depends on the oxoguanine dna glycosylase (OGG1) gene and 8-oxoguanine accumulates in the mitochondrial dna of OGG1-defective mice. Cancer Res.

[B13] Chevillard S, Radicella JP, Levalois C, Lebeau J, Poupon MF, Oudard S, Dutrillaux B, Boiteux S (1998). Mutations in OGG1, a gene involved in the repair of oxidative DNA damage, are found in human lung and kidney tumours. Oncogene.

[B14] Shinmura K, Yamaguchi S, Saitoh T, Kohno T, Yokota J (2001). Somatic mutations and single nucleotide polymorphisms of base excision repair genes involved in the repair of 8-hydroxyguanine in damaged DNA. Cancer Lett.

[B15] Audebert M, Chevillard S, Levalois C, Gyapay G, Vieillefond A, Klijanienko J, Vielh P, El Naggar AK, Oudard S, Boiteux S, Radicella JP (2000). Alterations of the DNA repair gene OGG1 in human clear cell carcinomas of the kidney. Cancer Res.

[B16] Shapiro RA, Skinner DG, Stanley P, Edelbrock HH (1984). Renal tumors associated with tuberous sclerosis: the case for aggressive surgical management. J Urol.

[B17] Stillwell TJ, Gomez MR, Kelalis PP (1987). Renal lesions in tuberous sclerosis. J Urol.

[B18] Al-Saleem T, Wessner LL, Scheithauer BW, Patterson K, Roach ES, Dreyer SJ, Fujikawa K, Bjornsson J, Bernstein J, Henske EP (1998). Malignant tumors of the kidney, brain, and soft tissues in children and young adults with the tuberous sclerosis complex. Cancer.

[B19] Walker C, Goldsworthy TL, Wolf D, Everitt J (1992). Predisposition to carcinogen-induced renal cell carcinoma due to alteration of a cancer susceptibility gene. Science.

[B20] McDorman KS, Wolf DC (2002). Use of the spontaneous Tsc2 knockout (Eker) rat model of hereditary renal cell carcinoma for the study of renal carcinogens. Toxicol Pathol.

[B21] Habib SL, Phan MN, Patel SK, Li D, Monks TJ, Lau SS (2003). Reduced constitutive 8-oxoguanine-DNA glycosylase expression and impaired induction following oxidative DNA damage in the tuberin deficient Eker rat. Carcinogenesis.

[B22] Habib SL, Riley DJ, Bhandari B, Mahimainathan L, Choudhury GG, Abboud HE (2007). Tuberin Regulates OGG1 in Renal Epithelial Cells and Kidney Tumors from Humans with Tuberous Sclerosis. Am J Physiol Renal Physiol.

[B23] Hemminki K, Koskinen M, Rajaniemi H, Zhao C (2000). DNA adducts, mutations, and cancer. Regul Toxicol Pharmacol.

[B24] Bradford MM (1976). A rapid and sensitive method for the quantitation of microgram quantities of protein utilizing the principle of protein-dye binding. Anal Biochem.

[B25] Hazra TK, Izumi T, Boldogh I, Imhoff B, Kow YW, Jaruga P, Dizdaroglu M, Mitra S (2002). Identification and characterization of a human DNA glycosylase for repair of modified bases in oxidatively damaged DNA. Proc Natl Acad Sci USA.

[B26] Barnes J, Mitchell R, Kanalas J, Barnes VL (1999). Differential expression of thrombospondin and cellular fibronectin during remodeling in proliferative glomerulonephritis. J Histochem Cytochem.

[B27] Tchou J, Bodepudi V, Shibutani S, Antoshechkin I, Miller J, Grollman AP, Johnson F (1994). Substrate specificity of Fpg protein. Recognition and cleavage of oxidatively damaged DNA. J Biol Chem.

